# Job Satisfaction and Hospital Performance Rated by Physicians in China: A Moderated Mediation Analysis on the Role of Income and Person–Organization Fit

**DOI:** 10.3390/ijerph17165846

**Published:** 2020-08-12

**Authors:** Xiao Chen, Min Liu, Chaojie Liu, Fang Ruan, Yan Yuan, Change Xiong

**Affiliations:** 1 School of Basic Medical Science, Hubei University of Science and Technology, Xianning 437100, China; xiaochen@hbust.edu.cn; 2 Department of Social Medicine and Health Management, School of Basic Medical Science, Hubei University of Science and Technology, Xianning 437100, China; c_xiong@hbust.edu.cn (M.L.); rf9916@hbust.edu.cn (F.R.); yuanyan89@hbust.edu.cn (Y.Y.); 3 School of Psychology and Public Health, La Trobe University, Melbourne, VIC 3086, Australia; c.liu@Latrobe.edu.au; 4 School of Public Health, Medical College, Wuhan University of Science and Technology, Wuhan 430065, China

**Keywords:** job satisfaction, person–organization fit, organizational performance, hospital

## Abstract

This study tested the effect of person–organization fit (P-O fit) in mediating the link between job satisfaction and hospital performance with income as a moderator. A questionnaire survey was conducted on 301 physicians from two public hospitals in Zhejiang province of China. Respondents were asked to rate their job satisfaction, value congruence (P-O fit) with the hospital, and the hospital’s performance. The mediating effect of P-O fit on the link between job satisfaction and hospital performance was tested through partial least squares-structural equation modeling (PLS-SEM). Income was introduced to the model as a moderator on the “P-O fit → hospital performance” and “job satisfaction → hospital performance” path, respectively. Higher job satisfaction and P-O fit were associated with higher ratings on hospital performance (*p* < 0.01). P-O fit had a partial mediating effect on the association between job satisfaction and hospital performance, accounting for 73% of the total effect. The effects of P-O fit and job satisfaction on hospital performance were stronger in the respondents with higher income. Overall, high job satisfaction is associated with high ratings on hospital performance, which is partially mediated through P-O fit. Value congruence is particularly important when financial tools are used to incentivize hospital physicians.

## 1. Introduction

Hospital management aims to achieve excellence in patient care through quality services delivered by highly motivated employees. Internationally, there is a shortage of healthcare workforce. Hospitals often struggle to retain highly skilled employees. Under such circumstances, hospital managers often see health workers as a core capital asset and use various measures to satisfy their needs in order to attract and retain them. Empirical evidence shows that higher job satisfaction can lead to lower turnover of hospital employees [1], which is associated with higher quality of patient care [2].

However, patient care services rely on team efforts and need to be delivered in a coordinated way. Organizational culture and management measures play a critical role in shaping the way in which health workers with different professional skills work together. This imposes a significant impact on the performance of hospitals [3,4]. Hartmann et al. [5] found that the prevalent hierarchical structure in hospitals is detrimental to patient safety. Research shows that a participatory approach to management may attenuate such an adverse effect [6]. 

The concept of performance is perhaps one of the most elusive ones in organizational theory [7]. Since the 1980s, the Chinese government has dramatically reduced budget support to public hospitals. Meanwhile, development of private hospitals has been encouraged. Such a reform has virtually pushed public hospitals to compete against each other and with the private sector. It was estimated that the government budget contributes less than 15% of the revenue of public hospitals. The survival, or otherwise, of a public hospital depends on its capability to attract patients and charge fees from patients and their insurance funds [8]. Indeed, many public hospitals were privatized in the 1990s. However, competitive positioning and market share of public hospitals have rarely been studied. Most existing studies on public hospitals simply assume that public hospitals would put patients at the center rather than chasing profits. Due to the complex nature of healthcare services, which often involve competing interests from multiple stakeholders, it is widely believed that hospital operations need to balance those interests. Sicotte et al. [7] summarized four approaches in examining hospital performance: rational/goal model, natural system model, open system model, and internal/decision process model. The rational model has a strong focus on explicit objectives. While the natural system model emphasizes internal health (such as morale, climate, cohesion, conflict, human development, and survival) of an organization, the open system model looks into external health (relationships with external stakeholders and success in acquisition of resources) of an organization. The internal process model assesses the effectiveness of operations management. Researchers have tried to integrate the four approaches [7,9,10]. In the health industry, the balanced scorecard metrics developed by Norton and Kaplan is perhaps the most widely used, which pulls together all of these aspects into one set of metrics [11,12]. Unfortunately, such a comprehensive set of metrics is neither available nor preferable for the purpose of this study. Hospital employees are less likely to hold conflict interests with their employers (hospitals) on matters beyond their controls, such as legal compliance requirements. They also share a strong expectation of delivering high-quality care. Inclusion of these indicators would not help, but conceal, potential conflicts between physicians and hospitals. In contrast, operations indicators are highly likely to expose the potential conflicts between physicians and hospitals. Staff remuneration is perhaps one of the most studied motivational aspects of staff satisfaction [13]. But in China, public hospitals are under tremendous pressure to save costs in order to achieve competitive advantage [6]. 

Studies have proven that job satisfaction is a strong predictor of organizational performance in a range of industries, including the health industry [14,15]. The underlying mechanisms of such a link remain unclear. A common understanding is that employees with high job satisfaction are likely to be more enthusiastic with their job tasks. However, hospitals are subject to pressures from multiple stakeholders and their interests are not always well-aligned with each other. For example, patients and their insurance funds demand on high-quality care with minimal costs, which may be contradictory to the financial interest of medical doctors in a fee-for-service payment system. Indeed, healthcare reforms, whether at an organizational level or at a national level, can significantly alter the view of employees on their hospitals. This has prompted an assumption of the importance of person–organization fit (P-O fit). A good P-O fit can drive individual efforts toward the expectations of the organization, which has been proven to be true in the nursing profession [16]. Hunt [17] believes that the value congruence of leadership support can motivate nurses to work harder. A good P-O fit enables a feeling of self-worthiness, creating a good working environment that encourages work effectiveness and organizational commitment of employees. 

Extensive studies have been undertaken to assess the fitness of a person into her/his working environment, such as with the job, with the team, with the organization, and with the profession, just to name a few [18]. According to Kristof [19], P-O fit is perhaps the most important one for the managers who try to maximize the value of human capital for the organization. It has attracted the most attention from the research community. The fitness can be examined from the perspectives of value, need, KSA (knowledge, skills, and abilities), goal, and personality [20]. Schneider’s Attraction–Selection–Attrition (ASA) theory suggests that value similarity influences the attractiveness of an organization to its employees [21]. The majority of the existing empirical studies have focused on the congruence between employee and organizational values. 

There is a paucity in the literature documenting the effect of P-O fit on hospital performance despite some studies on its impacts on employees, especially nurses [16,17]. It is important to note that occupational differences also exist between medical doctors and nurses in their responses to management measures [22]. This study aims to test the effect of P-O fit on hospital performance in a sample of hospital doctors in China based on the following hypotheses:

**Hypotheses** **1.** *P-O fit has a direct link with hospital performance.*

According to the P-O fit theory, work attitudes and performance of an employee are a reflection of actions for both personal and organizational purposes, rather than one of the sides separately [23]. A good P-O fit is beneficial in maintaining the loyalty and wellbeing of employees, which helps the development of positive work attitudes and behaviors of employees [16,24]. A systematic review and meta-analysis showed that P-O fit is associated with organizational citizenship behaviors and organizational commitment of employees [25]. Its impact on organizational outcomes is much stronger than on job-related outcomes [25,26]. 

It is challenging to study P-O fit of medical doctors, as in many health systems medical doctors tend to serve as a consultant rather than an employee. China provides an appropriate setting for such a study. Most medical doctors and nurses in China seek full-time and permanent employment in hospitals. Song and colleagues [27], in a study of nine public hospitals in China, found that P-O fit is positively associated with staff engagement and negatively associated with intention to leave in medical workers. Cui et al. [28] also found that P-O fit is positively correlated with self-efficacy of hospital nurses. We postulate that the positive effects of P-O fit on individual employees can eventually translate into better hospital performance.

**Hypotheses** **2.** *P-O fit mediates the effect of job satisfaction on hospital performance.*

In the management literature, there is abundant evidence to support the link of job satisfaction with individual performance, but less so for its link with organizational performance. Bakotić [29] found that job satisfaction determines organizational performance, although the intensity of the connection is weak. Scholars have proposed several theories to interpret the link, such as the motivation theory and the wellbeing and productivity theory [30]. Happy workers perform better than the “less happy” ones [31]. 

However, Ibrahim and Yusra [32] noted that individual satisfaction can sometimes put organizational goals at risk when the value of the organization is not endorsed by the employees. The Minnesota Work Adaption Theory [33] recommends that the relationship between an employee and her/his work environment can be considered as a process of mutual adaption. They influence and restrict each other. Individuals have a fundamental drive to fit in with their environment. They will strive to maintain a consistent relationship with their work environment when the environment satisfies their needs. When there is good value congruence, individual decisions will be easier. People will be happy to pursue the organizational values simply because these are also their personal values. However, if these two are not aligned, people may pursue their own interest. If the organizational goals deviate from the individual needs, a highly motivated employee may contribute little, if not jeopardize, the attainment of organizational goals. Indeed, psychological contract breach (PCB), a perception of organizational failure in fulfilling its obligations to employees, has become a frequent and even inevitable phenomenon in the constantly changing socioeconomic environment [34].

Health workers have been educated to put patient interest at the center of care. This may have led to a blank assumption that there is little conflict of interests between patients and hospitals and their employees. However, this is not necessarily true. Over the past few decades in China, there have been increasing debates about the perverse incentives imposed by the individual and organizational performance assessment measures introduced to public hospitals. Hospitals and medical doctors are blamed to make profits from over-provision of unnecessary services. Meanwhile, the dark side of management measures that promote high hospital performance has also started to attract attention from the research community. Several studies revealed that hospital management measures may pose heavy burdens on employees and even harm their physical and mental wellbeing [35,36]. Empirical studies proved that staff overloading is also a serious risk of patient safety [37]. This calls for a fine balance among the interests of various stakeholders. 

P-O fit may help achieve the balance of interests between hospitals and their employees. If there exists value congruence between the two parties, hard-working employees are more likely to feel appreciated and satisfied. A lack of P-O fit, on the other hand, may jeopardize the performance of an organization as a result of low levels of organizational commitment and loyalty from employees. Although there is a lack of studies into the mediating role of P-O fit in the relationship between job satisfaction and hospital performance, P-O fit has been found to be a mediator on the impacts of several management measures; for example, in the link of talent management with job satisfaction and organizational citizenship behaviors, in the interaction effect of high-performance work system (HPWS) and psychological contract breach (PCB) on employee engagement, and in the relationships between ethical culture and employee outcomes and between training and employee performance. 

Several studies have shown that the concept of P-O fit is applicable to the Chinese cultural context [28,38,39]. A previous study revealed that the effects of job satisfaction on organizational performance, whether financial or non-financial, are often indirect and mediated by perceived fairness and trust from employees [38]. This study responds to the call for research into the relationships between P-O fit, job satisfaction, and hospital performance in China. Song and colleagues [27] argue that this is particularly relevant to the latest health policy reform in China, in which increasing mobility of health workers is advocated.

**Hypotheses** **3.** *Income moderates the association between P-O fit and hospital performance.*

Organizations can take strategic actions to improve P-O fit using human resources and financial tools. Cui et al. [28] found that high levels of organization support are associated with perceived P-O fit. A relatively good income level is one of the most common type of organizational support. It is closely associated with job satisfaction.

In general, the Confucian culture encourages hierarchy, collectivism, and a rejection of self-centeredness [40,41]. Like many other countries, the medical profession occupies the highest rank in the hierarchical structure of hospitals in China. Medical doctors in China appear to enjoy an even higher status in decision making as these decisions not only determine patient care outcomes, but are also linked to the financial prospect of hospitals. Over the past few decades, government budgets providing support to public hospitals have been substantially reduced. Meanwhile, hospitals are increasingly exposed to the market forces. This has created a dilemma. On one hand, public hospitals have been subject to strict restrictive measures from the government (e.g., zero markup on sales of medicines) for concerns of affordability of care. On the other hand, medical doctors have been forced to develop coping strategies to protect the financial interests of their hospitals. As a result, income has become one of the most important instruments in China to incentivize medical doctors in hospitals, despite the fact that there is limited evidence to support a link between high renumeration and quality of patient care. A low-salary and high-bonus system was established in public hospitals in China. In such a system, bonuses are inevitably linked to the financial revenue of the hospital and the contribution made by each individual employee [8]. It has been criticized for increasing the chance of moral hazards from medical workers and eroding trust from the government and consumers. 

In the management literature, income is usually considered as a “hygiene” factor. Low-pay can cause job dissatisfaction, but high-pay is not necessarily a motivator that creates job satisfaction. A meta-analysis concluded that job satisfaction is only marginally related to pay levels [13]. Monetary incentives may also have some detrimental effects on individual performance of the tasks that require high cognitive skills. In this study, we hypothesized that income would not change the positive link between job satisfaction, P-O fit, and hospital performance. However, the extent of such links is moderated by income.

## 2. Materials and Methods

A cross-sectional survey was conducted on 301 physicians in two tertiary public hospitals. Ethics approval (No.: 2016-09-007) for the study protocol was obtained from Hubei University of Science and Technology.

### 2.1. Study Setting and Sampling

The study was undertaken in Zhejiang province of China. Zhejiang is located in eastern China, with a population of 56.57 million. It ranks in the high range of economic development among all provinces of China in terms of per capita GDP. Zhejiang province consists of 11 municipalities. Two of the municipalities, Hangzhou (the capital city) and Ningbo, were conveniently selected. There were nine tertiary public hospitals in Hangzhou, compared with four in Ningbo. One public tertiary hospital from each of the two municipalities were used in this study.

### 2.2. Sampling and Participants

Data were collected from October 2016 to July 2017. Four investigators were recruited from Hubei University of Science and Technology and trained to conduct the survey through face-to-face interviews. All of the physicians on duty on the day when the investigators visited the sample hospitals were approached and invited to participate in the survey. In total, 450 invitations were dispatched and 301 (67%) physicians accepted and completed the survey. This represented 33% of all physicians (*N* = 916) employed by the two hospitals. The interviews were conducted in the office of the participating physicians. The investigators were unknown to the participants and no servicing relationship existed between the two. The participants were required to read the informed consent letter on the cover page before proceeding to the questionnaire. They were advised to imply their informed consent by returning the questionnaire voluntarily and anonymously.

### 2.3. Measures

This survey comprised three scales: (1) person–organization fit (P-O fit), (2) job satisfaction, and (3) hospital performance.

#### 2.3.1. P-O Fit

In this study, the instrument developed by Cable and Judge [42] was adapted to measure P-O fit, which was defined as perceived value congruence between individuals and organizations. A Chinese version of the scale has been validated [43]. It contains seven items measuring perceptive fitness of values between an individual and the organization. Respondents were asked to rate their value fitness with the hospital on a five-point Likert scale, ranging from 1 (not at all) to 5 (completely). An average summed score was calculated, with a higher score indicating higher P-O fit. 

P-O fit can be measured using a subjective or objective approach. The subjective measures capture individual perceptions about the extent to which they feel like they fit into their organization. In contrast, the objective measures calculate the similarity between the characteristics of an individual and an organization. Previous studies have shown that objective P-O fit is more strongly related to behavioral outcomes of employees [25]. However, there is a lack of consensus on the configuration of objective measures for values, especially in the hospital setting. The concept of value indicates the importance that people attach to things, which by itself is inherently subjective [44]. Therefore, a subjective measurement of value P-O fit serves better for the purpose of this study.

#### 2.3.2. Job Satisfaction

Kalleberg [45] defined job satisfaction as the affective attitudes of an employee toward her/his work after balancing various aspects of the work. This study adopted the scale developed by Tsui and colleagues [46], measuring job satisfaction based on Kalleberg’ s definition. The scale has been validated [47], including in studies in China [48]. It contains six items, evaluating aspects of the work itself, personal responsibility, colleagues, superiors, remuneration, and promotion. Respondents were asked to rate their satisfaction on a five-point Likert scales, ranging from 1 (strongly disagree) to 5 (strongly agree). An average summed score was calculated, with a higher score indicating higher job satisfaction.

#### 2.3.3. Hospital Performance 

In this study, participants were asked to rank their hospital performance against their regional competitors on a quintile scale ranging from 1 (bottom 20%) to 5 (top 20%). Seven items were adapted from the instrument developed by Tan and Litschert [48,49] measuring the financial prospect (four items measuring profit, sales, growth of sales, and growth of assets), market share (one item), employee morale (one item), and competitive positioning (one item) of the hospitals. This instrument has been validated in China [50]. An average summed score was calculated, with a higher score indicating higher hospital performance.

This study adopted the Tan and Litschert instrument to evaluate hospital performance for two reasons. Firstly, this instrument emphasizes financial operations, which are closely associated with remunerations of employees in public hospitals in China. Secondly, this instrument adopts a self-rating approach. Tan and Litschert [49] suggest that employees are well positioned to compare the relative performance of their organization with its close competitors. Previous studies have proven that perceptive performance can serve as a useful alternative measures of objective performance [51]. Compared with objective performance data, self-rating can better reflect perceived individual contributions of an employee to the hospital. Hospital performance is a result of collective efforts and individuals are not necessarily able to contribute to all aspects of the hospital. Perceptive measurements build a natural connection between individual employees and hospital performance. 

### 2.4. Statistical Analysis

Data were analyzed using SPSS 26.0 and Smart PLS 3.0 (SmartPLS GmbH, Boenningstedt, Germany). A *p*-value less than 0.05 was considered statistically significant.

Partial least squares-structural equation modeling (PLS-SEM) was performed to test the mediating effect of P-O fit on the link between job satisfaction and hospital performance. PLS-SEM is known as a second-generation regression technique for complex causal modeling. We followed a two-step approach. The first step assessed the reliability of the measurements through Cronbach’s alpha (CA; CA > 0.7), composite reliability (CR > 0.7), and average variance extracted (AVE; AVE > 0.5), and the convergent validity of the measurements using the Fornell–Lacker criterion on item loadings [52]. A loading above 0.70 is preferable [53]. According to Hair et al., a minimum reliability of 0.6 is required for an exploratory study, while research using established measures should have a reliability of at least 0.7 [54]. However, the items with a loading below 0.70 but above 0.40 should be removed only if the removal results in an increase in the composite reliability. An acceptable discriminatory validity is demonstrated by a higher square root of AVE for each measurement scale in comparison with its correlation coefficients with other scales. The second step assessed the statistical significance of the path coefficients. A bootstrapping with 5000 samples was used to estimate the path coefficients. A Q^2^ value greater than zero generated by a blindfolding procedure verifies predictive relevance of the structural model [54].

The average monthly income of the study participants was divided into two categories: low-income (≤¥5000 yuan) and high-income (>¥5000 yuan). In both low- and high-income groups, a linear correlation was found between job satisfaction and hospital performance and between P-O fit and hospital performance. The slope of the correlation lines in the high-income group was greater than that of the low-income group (Appendix A). Therefore, income was introduced into the PLS-SEM as a moderator on the path “P-O fit → hospital performance” and “job satisfaction → hospital performance”, respectively. We also analyzed the moderating effect of work experience on these paths to test the potential of income serving as a proxy indicator of work experience.

Two items measuring job satisfaction had a loading between 0.4 and 0.7, while the rest were all above 0.70. We performed sensitivity tests comparing the PLS-SEM results between those exclusive of the two items and those without exclusive of these items. The results were consistent (Appendix B).

## 3. Results

### 3.1. Characteristics of Respondents and Their Ratings on Job Satisfaction, P-O Fit, and Hospital Performance

More than 89% of the 301 respondents were under 50 years old. Most were female (61%), younger than 40 years (53%), obtained a highest qualification of a bachelor’s degree (55.82%), and had a junior professional title (69%). The majority (58%) earned a monthly income below ¥5000 Yuan. About 55% had a work experience between one and five years (Table 1). 

On average, the respondents had a score of 3.22 (SD = 0.76), 3.28 (SD = 0.88), and 2.98 (SD = 1.10) out of a maximal possible of 5 for job satisfaction, P-O Fit, and hospital performance ratings, respectively. Significant differences in hospital performance ratings were found in the respondents with different sociodemographic characteristics, however, no significant differences in job satisfaction and P-O fit were found in those with different age and professional titles. The respondents with a higher income had a higher score in job satisfaction (F (2.298) = 18.55, *p* < 0.001), P-O fit (F = 31.36, *p* < 0.001), and hospital performance ratings (F = 37.37, *p* < 0.001). The male respondents had higher scores in all the three measurements than their female counterparts. Lower scores in the three measurements were also associated with longer years of work experience (Table 1). 

### 3.2. Reliability and Validity of Measurement Scales

Good reliability was demonstrated in the three measurement scales according to the pre-defined criteria (Table 2). All had a higher than 0.5 AVE and greater than 0.7 Cronbach’s α and composite reliability coefficients.

The square root of AVE for each scale is consistently greater than its correlations with other scales, indicating acceptable discriminatory validity (Table 3).

### 3.3. PLS-SEM Results

The (unreported) variance inflation factor (VIF) of the constructs of the predictors was below 3.0, indicating the absence of collinearity. The Q^2^ of the structural model was above zero, which verified the predictive relevance of the structural model. 

**Figure 1:**
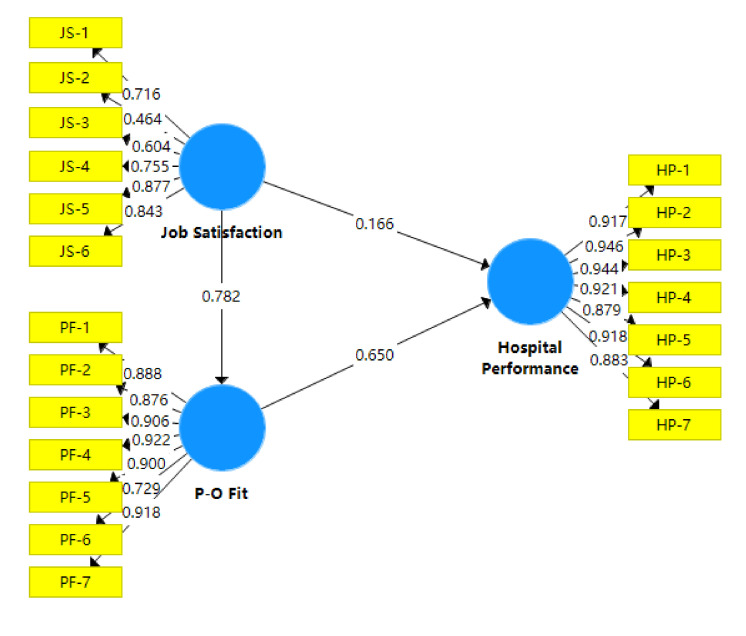
Mediating effect of P-O fit (Person-Organization fit) on the link between job satisfaction and hospital performance.

The PLS-SEM results showed that job satisfaction predicted (Q^2^ = 0.365) and explained 47.9% of the variance of hospital performance (R^2^ = 0.479, R^2^ deviation from zero), with a path coefficient of 0.692 (*p* < 0.001). The adding of P-O fit as a mediating factor reduced the path coefficient to 0.166 (Figure 1 and Table 4), but still with statistical significance (*p* < 0.01). P-O fit was a significant predictor of hospital performance, which absorbed 75.37% of the effect of job satisfaction on hospital performance as measured by the variance accounted for (VAF). The direct effect between job satisfaction and hospital performance remained significant (*t* = 2.856, *p* = 0.004). 

**Figure 2:**
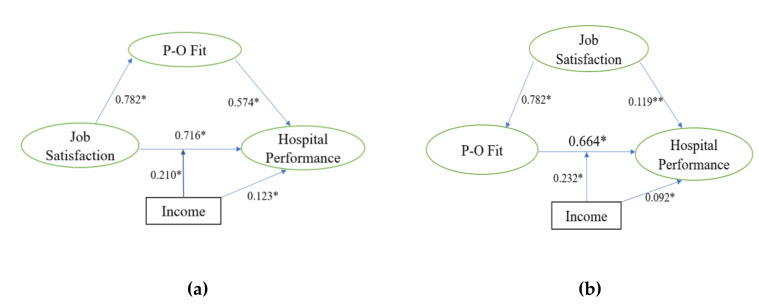
Income as a moderator on the effects of job satisfaction and P-O fit (Person-Organization fit) on hospital performance. (**a**) Moderating effect of income on “Job satisfaction → Hospital performance”: *t* = 4.080, *p* < 0.001. * *p* < 0.05. (**b**) Moderating effect of income on “P-O fit → Hospital performance”: *t* = 3.693, *p* < 0.001. * *p* < 0.05, ** *p* < 0.01.

Income was confirmed to be a moderator over the path between job satisfaction and hospital performance and between P-O fit and hospital performance (Figure 2), supporting hypothesis three. All of the path coefficients remained statistically significant (*p* < 0.05). The association between job satisfaction and hospital performance was stronger in those with high-income. Similarly, a stronger association between P-O fit and hospital performance was also found in those with high-income.

Further analyses showed that gender had no direct effect on hospital performance ratings. Instead, it moderated the link between job satisfaction and hospital performance. In contrast, work experience was associated with hospital performance ratings. However, it did not have a moderating effect in the SEM (Appendix C).

## 4. Discussion

The three hypotheses were supported in this study. P-O fit was proven to have a positive effect on hospital performance as perceived by the hospital doctors (H1). The effect of job satisfaction on hospital performance was mediated by P-O fit (H2). Income was a moderator for the link between P-O fit and hospital performance and the link between job satisfaction and hospital performance (H3). The link between P-O fit and hospital performance was stronger in those with high-income.

### 4.1. Main Findings

This study confirmed the mediating role of P-O fit in the link between job satisfaction of doctors and hospital performance. It also adds some new insight into the role of renumeration. Internationally, human resource managers in hospitals have been struggling with retaining and motivating highly skilled health professionals. Although this study was conducted in China, it can offer some lessons to other countries with a similar health reform agenda, in particular in terms of the use of financial and market tools to drive high performance of hospitals. 

Job satisfaction is an intrinsic motivator of highly skilled health professionals. The positive link between job satisfaction and hospital performance revealed in this study is consistent with findings of previous studies [55,56,57]. Common strategies adopted by managers to improve job satisfaction include management and resource support, work-life balance, and teamwork. Autonomy has been highly valued in health professions and it has a positive link with job satisfaction. Historically, medical doctors have enjoyed a high level of autonomy in clinical decisions. Clinical autonomy is believed to be critical to ensure evidence-based practices. In a rapidly changing socioeconomic environment with an explosion of knowledge production, clinical autonomy demonstrates the commitment of health organizations to put patient interests at the center of care. Insufficient of clinical autonomy can result in a dispirited, insecure, and unconfident medical workforce. However, empirical evidence shows that the behaviors of medical doctors are not only driven by their clinical knowledge and expertise, they are also shaped by the external environment in which they work [58]. This has highlighted the importance of aligning the work environment with the needs of medical doctors. The current study provides further evidence to support such an argument. It proves that P-O fit is not only positively linked with hospital performance as it also mediates the link between job satisfaction and hospital performance. 

The positive link between P-O fit and organizational performance has been established in a few studies [16]. P-O fit in value was found to be able to reduce the cognitive conflicts between physicians and hospitals. Value shapes the choice and motivation of individual actions. A good P-O fit in value is likely to be recognized by employees as an indication of organizational appreciation. It may alleviate the work-related conflicts that demotivate employees. Unfortunately, public organizations often pay little, if any, attention to the individual values held of their employees [16]. The findings of this study imply that the P-O fit theory is applicable to the public hospital setting in China. We found that P-O fit has a much stronger link with hospital performance than job satisfaction. In addition, P-O fit can explain 75% of the effect of job satisfaction on hospital performance. 

The management consideration of using renumeration to incentivize staff for better performance deserves some debates. This study demonstrated a greater moderating effect of income on hospital performance through job satisfaction and P-O fit in comparison with its direct link to hospital performance. However, the direct effect of income on hospital performance ratings remained statistically significant. The income effect is unlikely to be a confounding effect of work experience as work experience is not a significant moderator for the effects of job satisfaction and P-O fit on hospital performance. In China, basic salary levels are generally low. Hospital managers tend to use bonuses to incentivize their employees, which is usually linked to work outputs rather than work experience. The low-income of health workers has been a serious challenge in many developing countries. The findings of this study highlight the important role of income, although an increase in income by itself is not enough. Managerial efforts to avoid perverse financial incentives need to be emphasized. These may include strengthening of P-O fit in value.

The importance of employees in gaining competitive advantage has been recognized by many organizations, including the WHO [59]. However, it is insufficient to concentrate only on the wellbeing and satisfaction of hospital employees. Managers need to realize that P-O fit is an effective mechanism to harmonize the interests between employees and the organization. This study shows that P-O fit is even more important when hospital doctors are rewarded with higher pay. There are several strategies to improve P-O fit, such as selective recruitment, social and cultural activities, and staff training and development. Strong leadership commitment and strategic planning is essential. There is evidence that job satisfaction can enhance a sense of person–organization fit in employees within a hierarchical structure [1,60].

This study makes some theoretical contributions. The link between job satisfaction and individual and organizational performance in the health industry has started to attract increasing attention in the literature [56]. However, research into the underlying mechanisms of such a relationship is quite limited, in particular in relation to the role of P-O fit, a concept that is often taken for granted in the public hospital sector. This study fills the gap in the literature. The study proved that P-O fit is likely to be an important mediating factor for management measures that look after the wellbeing of hospital employees. The link revealed in this study also shows off the high statistical power of PLS-SEM and its use on theory testing and confirmation. We advocate for further studies into the potential mediating effects of P-O fit on the link between a set of human resource management measures under the umbrella of a high-performance work system (HPWS) and patient care outcomes. 

### 4.2. Limitations

This study has several limitations. It adopted a cross-sectional design. No causal relationships should be assumed. The study sample was drawn from two public hospitals in China, which is not representative of the entire hospital sector in China. 

## 5. Conclusions

High job satisfaction of hospital doctors is associated with their high ratings on hospital performance, which is partially mediated through P-O fit. The mediating function of P-O fit explains more than 75% of the effect of job satisfaction on hospital performance. Value congruence is particularly important when financial tools are used to incentivize hospital doctors. The link between job satisfaction, P-O fit, and hospital performance will become stronger when hospital doctors are rewarded with higher pay. 

## Figures and Tables

**Table 1:** Sociodemographic characteristics and measurement (job satisfaction, P-O fit, hospital performance) scores of study participants.

Characteristics		*n*	%	Mean ± Standard Deviation (SD)		
				Job Satisfaction	P-O Fit	Hospital Performance
Gender	Male	117	38.87	3.50 ± 0.79	3.55 ± 0.82	3.31 ± 1.09
	Female	184	61.13	3.26 ± 0.66	3.10 ± 0.87	2.76 ± 1.05
				*p < 0.001*	*p < 0.001*	*p < 0.001*
Age (Years)	30–39	161	53.49	3.28 ± 0.76	3.33 ± 0.85	3.07 ± 1.04
	40–49	101	33.55	3.19 ± 0.68	3.27 ± 0.90	2.97 ± 1.15
	50–59	39	12.96	3.04 ± 0.93	3.08 ± 0.93	2.58 ± 1.14
				*p = 0.182*	*p = 0.297*	*p = 0.041*
Educational attainment	Bachelor’s degree	168	55.82	3.20 ± 0.80	3.14 ± 0.93	2.72 ± 1.13
	Postgraduate degree	133	44.18	3.26 ± 0.70	3.45 ± 0.78	3.30 ± 0.97
				*p = 0.493*	*p < 0.001*	*p = 0.002*
Monthly income (Yuan)	2000–5000	175	58.14	3.01 ± 0.71	2.97 ± 0.87	2.60 ± 0.96
	5001–10000	85	28.24	3.46 ± 0.77	3.61 ± 0.76	3.27 ± 1.11
	≥10000	41	13.62	3.62 ± 0.66	3.87 ± 0.52	3.97 ± 0.80
				*p < 0.001*	*p < 0.001*	*p < 0.001*
Working experience (Years)	1–5	166	55.15	3.36 ± 0.76	3.44 ± 0.85	3.27 ± 1.04
	6–10	67	22.26	3.18 ± 0.67	3.19 ± 0.92	2.79 ± 1.70
	11–20	68	22.59	2.94 ± 0.77	2.96 ± 0.82	2.44 ± 0.92
				*p = 0.001*	*p < 0.001*	*p < 0.001*
Professional title	Junior	209	69.44	3.28 ± 0.75	3.36 ± 0.86	3.11 ± 1.09
	Intermediate	68	22.59	3.13 ± 0.67	3.09 ± 0.87	2.74 ± 1.06
	Senior	24	7.97	3.01 ± 1.04	3.09 ± 1.03	2.50 ± 1.06
				*p = 0.119*	*p = 0.054*	*p = 0.005*

**Table 2:** Reliability of measurement scales.

Construct		Loading	Cronbach’s α	Composite Reliability	AVE
Job satisfaction			0.827	0.864	0.524
JS-1	With promotion opportunity	0.716			
JS-2	With colleagues	0.464			
JS-3	With immediate superior	0.604			
JS-4	With individual tasks	0.755			
JS-5	With remuneration	0.877			
JS-6	Overall	0.843			
P-O fit			0.951	0.960	0.773
PO-1	Personal value in organizational value	0.888			
PO-2	Personal value in organizational culture	0.876			
PO-3	Personal life in organizational value and culture	0.906			
PO-4	Person–organization match	0.922			
PO-5	Person–organization value match	0.900			
PO-6	Person–colleague value match	0.729			
PO-7	Personality fit into organization	0.918			
Hospital Performance			0.968	0.973	0.838
HP-1	Profitability	0.917			
HP-2	Annual revenue	0.946			
HP-3	Market share in revenue	0.944			
HP-4	Revenue growth	0.921			
HP-5	Staff morale	0.879			
HP-6	Net assets	0.918			
HP-7	Competitive positioning	0.883			

**Table 3:** Square root of AVE and Pearson correlation coefficients of measurement scales.

Variable	Job Satisfaction	P-O Fit	Hospital Performance
Job Satisfaction	0.724		
P-O Fit	0.782	0.879	
Hospital Performance	0.674	0.780	0.916

Note: Bold and diagonal values represent the square root of AVE whereas the off diagonals represent the correlations of constructs.

**Table 4:** Structural model assessment (PLS path model with mediator).

Path	Coefficient	Bias Corrected 95%	Confidence Intervals	*t* *	*p*
P-O fit → Hospital performance	0.650	0.551	0.751	12.639	<0.001
job satisfaction → P-O fit	0.782	0.736	0.825	33.992	<0.001
job satisfaction → hospital performance	0.166	0.051	0.277	2.856	0.004

P-O fit: R^2^ = 0.611, Q^2^ = 0.426; Hospital performance: R^2^ = 0.619, Q^2^ = 0.471. * *t* values of independent sample *t* tests.

**Figure A1:**
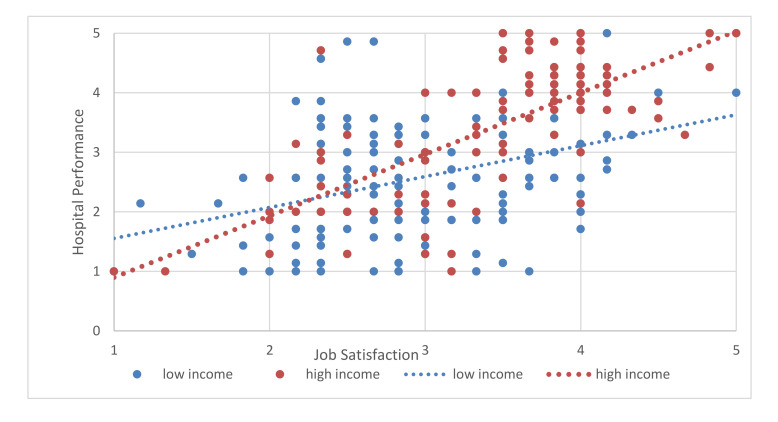
Correlation between job satisfaction and hospital performance categorized by income.

**Figure A2:**
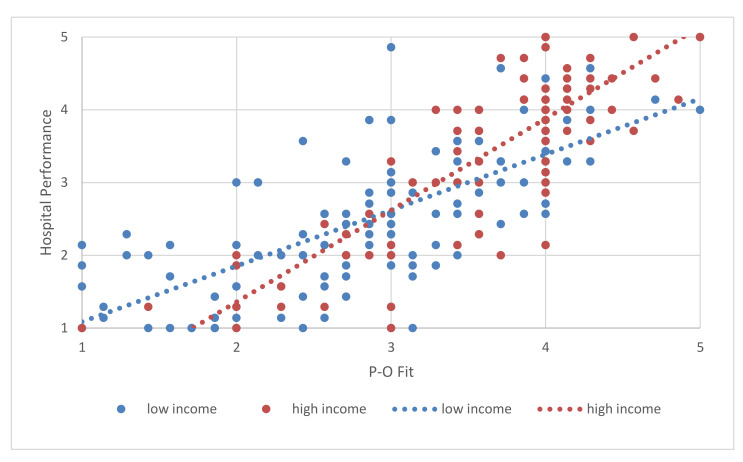
Correlation between job satisfaction and hospital performance categorized by income.

**Figure A3:**
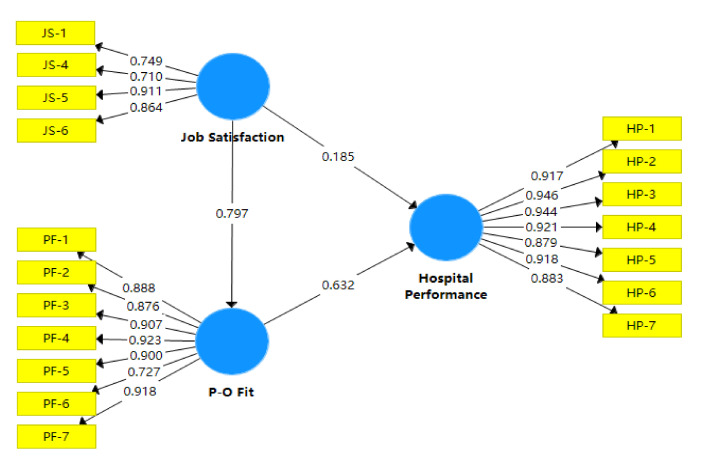
Mediating effect of P-O fit (Person-Organization fit) on the relationship between job satisfaction and hospital performance (Two items deleted from job satisfaction scale).

**Figure A4:**
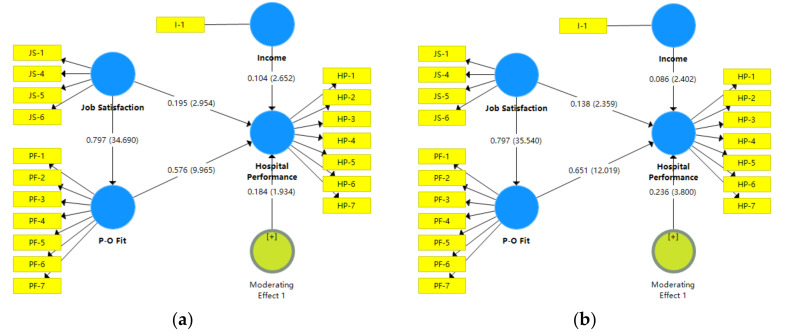
Moderating effects of income (two items deleted from job satisfaction scale). (**a**) Moderating effect of income on “Job satisfaction-Hospital performance”: *t* = 1.934, *p* = 0.053; (**b**) Moderating effect of income on “P-O Fit -> Hospital performance”: *t* = 3.800, *p* < 0.001.

*Note*: P-O Fit = Personal-Organization Fit.

**Figure A5:**
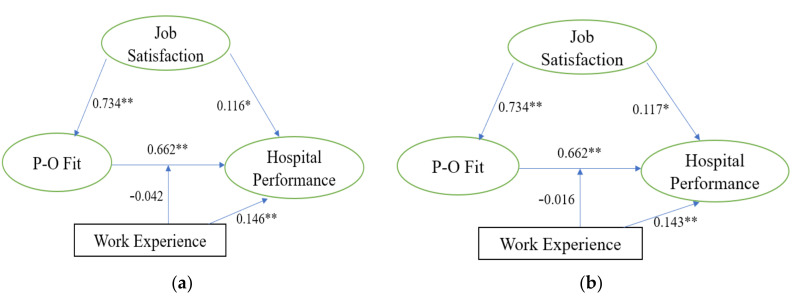
Work experience as a moderator for the effects of job satisfaction and P-O fit (Personal-Organization fit) on hospital performance. (**a**) Moderating effect of work experience on “P-O fit -> Hospital performance”: *t* = 1.136, *p* = 0.198; (**b**) Moderating effect of work experience on “Job Satisfaction-> Hospital performance”: *t* = 0.593, *p* = 0.553.

## Appendix A. Moderating Effect of Income

**Table A1:** Measurement scores (Mean ± SD) in the study participants with high (>¥5000 yuan) and low (≤¥5000 yuan) income.

Monthly Income (¥)	P-O Fit	Job Satisfaction	Hospital Performance
Low Income (n = 175)	2.97 ± 0.87	3.01 ± 0.71	2.60 ± 0.96
High Income (n = 126)	3.70 ± 0.70	3.51 ± 0.74	3.50 ± 1.07
*t*	−7.715	−5.97	−7.65
*p*	<0.001	<0.001	<0.001

## Appendix B. Sensitivity Tests

**Table A2:** Square root of AVE and Pearson correlation coefficients of the measurement scales(two items deleted from job satisfaction scale).

Variable	Job Satisfaction	P-O Fit	Hospital Performance
Job satisfaction	0.813		
P-O Fit	0.797	0.879	
Hospital performance	0.689	0.780	0.916

**Table A3:** Structural model assessment (PLS path model with mediator).

Path	Coefficient	Bias Corrected 95% Confidence Intervals	*t **	*p*
P-O Fit -> Hospital performance	0.632	0.525–0.751	11.17	<0.001
job satisfaction -> P-O Fit	0.797	0.754–0.835	36.85	<0.001
job satisfaction -> hospital performance	0.185	0.053–0.300	2.97	0.003

P-O Fit: R^2^ = 0.635, Q^2^ = 0.442. Hospital performance: R^2^ = 0.618, Q^2^ = 0.472. * *t* values of independent sample t tests.
